# PROTOCOL: The effectiveness of wilderness therapy and adventure learning in reducing anti‐social and offending behaviour in children and young people at risk of offending

**DOI:** 10.1002/cl2.1270

**Published:** 2022-08-31

**Authors:** Ashima Mohan, Suchi Malhotra, Monisha Narayanan, Howard White, Hannah Gaffney

**Affiliations:** ^1^ Campbell South‐Asia New Delhi India; ^2^ Campbell Collaboration Vasant Kunj India; ^3^ University of Cambridge Cambridge UK

## Abstract

This is the protocol for a Campbell systematic review. The objectives are as follows. The review will address the following research questions: (1) What are the long‐ and short‐term effects of wilderness therapy and adventure learning on anti‐social behaviour and violent and other offending behaviour? What factors explain any heterogeneity (i.e., moderate) these effects. What are the long‐ and short‐term effects of wilderness therapy and adventure learning on intermediate mental health and behaviour outcomes such as social skills and self‐regulation? What factors explain any heterogeneity (i.e., moderate) these effects? Factors such as setting (indoor/outdoor), quality of relationship with counsellors and the degree of the challenge element involved are important moderators of these effects, and help explain any observed heterogeneity across studies (2) What are the barriers and facilitators affecting the successful implementation of wilderness therapy and adventure learning programmes? (3) Are wilderness therapy and adventure learning interventions cost effective?

## 1 BACKGROUND

### 1.1 The problem, condition or issue

The use of challenges for positive youth development goes back at least a century, with John Dewey proposing learning through stressful tasks (Brendtro & Strother, 2007). Challenges are group‐based activities with tasks such as a group climbing over a high wall with limited equipment, constructing a bridge crossing, or building an access ramp for wheelchairs and pushchairs. One specific form of challenge which has become common are outward bound courses and wilderness therapy.

Outward bound course began in the United States in the early 1960s, and was specifically designed for teenagers from disadvantaged backgrounds and those displaying problem behaviours. Wilderness therapy was a further elaboration of the outward bound concept, which was developed in response to the growing demand for rehabilitation programs for youth with problem behaviours during the 1960s and 1970s (Kelly & Baer, 1968; Stewart, 1978). It provided an innovative treatment alternative for children and adolescents facing multiple challenges (Behar & Stephens, 1978). More recently the term adventure learning has been used for intervention in which children and young people learn skills through challenge activities.^1^

These groups of activities have three common components: group working, creating a safe space which can develop relationships of trust, overcoming a challenge, with a fourth component in some interventions of exposure to wilderness or nature. They may include a therapeutic component, but that is not required to be within the scope of this review.

Adolescents with behavioural challenges or disorders and who are at risk of anti‐social and offending behaviour have difficulty in adapting to social norms. Wilderness therapy and adventure learning may address these issues in various ways: the learning and self‐worth from the challenge element, developing pro‐social attitudes from activities requiring team‐work, and the therapeutic effects of creating safe spaces and being exposed to nature.

Wilderness therapy is rooted in hands‐on learning and draws from experiential education, that is ‘learning by doing’ along with reflection (Gass, 1993). Experiential education is a philosophy of education that ‘informs many methodologies in which educators purposefully engage with learners in direct experience and focused reflection in order to increase knowledge, develop skills, clarify values, and develop people's capacity to contribute to their communities’.^2^

Individuals or groups are placed in real‐life settings where they must use problem‐solving to deal with the environment and the work at hand in adventure programmes. (Hans, 2000). The different forms of adventure learning include wilderness treatment, adventure‐based activity learning, and long‐term residential camping (Gillis & Thomsen, 1996).

Wilderness and adventure interventions offer excitement and perceived risk to children who offend, which meets the desire for high arousal (Kelly & Baer, 1968). The group experience provided by wilderness therapies and challenge activities is appropriate for adolescents' developmental needs. It promotes peer relationships, emphasises community collaboration, and offers opportunity for the development of trust, effective communication, and problem‐solving abilities (Zimring, 1983).

Nature's potentially positive effect on human health has led nature‐based interventions to be proposed for improving both physical and mental health. In the case of at‐risk youth, wilderness and adventure learning programs are claimed to facilitate development of appropriate social and adaptive behaviours (Shanahan, 2019). Therapeutic benefits of wilderness programs have focused primarily on the positive changes in the participant's self‐esteem and self‐worth, as well as the development of pro‐social behaviours from group activities (Cook, 2008).

Existing studies suggest that outdoor and experiential education, wilderness therapy programs and challenge activities increase participants’ self‐esteem (Bowen & Neill, 2013; Wilson & Lipsey, 2000), and the belief that they have control over events that affect them (Hans, 2000; Wilson & Lipsey, 2000).

There is also evidence that outdoor education and experiential education programs with a longer duration have stronger effects than shorter programs (Sibthorp et al., 2007). Another evaluation of a wilderness therapy program for Canadian young offenders found that a 20‐day program has greater effects on social skills and motivation than a 10‐day program (Paquette & Vitaro, 2014). However, success of some other wilderness therapy programs does not appear to be affected by program duration (Wilson & Lipsey, 2000).

It is over 20 years since the publication of the last effectiveness review of wilderness therapy (Wilson & Lipsey, 2000), with a considerable number of studies published since then. There is a more recent review of outdoor challenge activities (Bowen & Neill, 2013), but it does not report offending outcomes. Given the continued interest in wilderness therapy, and the related area of adventure learning, and the current focus on youth crime, it is proposed to conduct a new, integrated mixed method review.

### 1.2 The intervention

The review will include wilderness therapy and adventure learning programs.

Wilderness programmes are defined as follows:

(1) take place in ‘wilderness’ or nature setting;
(2) have an overnight stay element; and
(3) have an interpersonal element which may include group activities, or work with counsellors and therapists.

Adventure learning involves challenge‐based activities in which children and young people, usually in a group, have to overcome a challenge. The challenge may be in an outdoor setting—but need not be wilderness, it could be a local park—but may also be indoors. The challenge is intended to bring about change at a meta‐process level (behaviours, cognitions and unconscious processes that impede or support therapeutic change) (Itin, 2001).

In both cases the intervention must be targeted at youth who are at risk of offending, which includes those who have already offended. That is, we will include only secondary and tertiary interventions.

The intervention may take place in any setting (custody, community or school).

### 1.3 How the intervention might work

Wilderness therapy and adventure learning programmes are designed to help the participants develop confidence in their abilities to accomplish difficult goals by involving them in a series of challenging tasks, usually undertaken as a group, hence teaching them communication and cooperation skills which develops a sense of trust.

The positive effects of wilderness and adventure learning can operate through a number of channels or causal processes. These are: (i) a ‘wilderness effect’ (nature); (ii) group activities encouraging pro‐social behaviour; (iii) developing self‐worth through completing challenges; (iv) diversion (i.e., spending time away from circumstances which may lead to anti‐social behaviour and offending), (v) the benefits of the counselling or therapeutic component which may happen formally or informally; and (vi) facilitation and the mentoring relationship with the counsellor. In addition to these factors, some practical aspects of programme design and implementation, such as duration and follow up arrangements, may also moderate programme effects. Each of these potential causal mechanisms is now discussed in turn.

#### 1.3.1 The wilderness effect

Setting interventions in relatively remote wilderness settings may have an effect through three possible channels: (i) the therapeutic effects of nature; (ii) isolated settings making it harder to dropout; and (iii) the unfamiliar setting reinforcing group bonding. Each of these channels is discussed briefly in turn.

There is a growing body of evidence that exposure to nature has positive therapeutic effects. Two recent systematic reviews report a positive association between nature‐based recreation and mental health (Lackey et al., 2021; and Tillmann et al., 2018). Hence, the outdoor setting of the wilderness program offers restorative benefits from exposure to nature. It may also utilize adolescents’ inclinations towards spontaneity and self‐disclosure (Hill, 2007; Russell & Farnum, 2004).

Children and Young People are referred to wilderness programmes, for example, by Youth Offending Teams in England, or as part of a diversion programme in the United States. But participation may be voluntary, so participants may drop out at any time. But doing so is a bigger deal when already having committed to a multi‐day activity in a remote location, rather than say a local activity centre with good public transport connections. Hence wilderness settings may encourage compliance. Preventing dropout may also be supported by preparation activities before the wilderness component.

As discussed below, undertaking a group challenge can assist bonding. Doing so in an unfamiliar environment, such as a wilderness setting, may in itself facilitate bonding. It may also facilitate the relationship with the counsellor as the young person depends on a ‘guide’ in an unfamiliar setting.

These various reasons for a ‘wilderness effect’ suggests that activities based in natural, remote settings may have stronger effects than otherwise similar programmes in an indoor setting, so that the setting (outdoor/indoor) may be an important moderator.

#### 1.3.2 Group activities and safe spaces to encourage pro‐social behaviour

Wilderness therapy programmes and adventure learning generally include group activities, which may include a challenge element such as finding their way back to base, rock climbing or using equipment to cross a river. Engaging in task‐oriented adventure activities for groups to solve as a single unit supports pro‐social behaviours and problem solving.

The activities may also provide a framework for the creation of a safe space. Youth may be more likely to open up in a structured activity where the focus is on a challenge than if they are sat if a circle and told ‘tell us about your feelings’. The design of wilderness therapy programs addresses communication difficulties among adolescents in this way (Fletcher & Hinkle, 2002). Group engagement in a structured setting are instrumental in overcoming adolescents’ difficulties that stem from limited verbal abilities, emotional and cognitive openness, and sharing personal thoughts (Hill, 2007).

The cohesion of the group is believed to be reinforced by the challenging nature of the activity (e.g., Glass & Benshoff, 2002). Being placed in an unfamiliar environment, combined with the challenge element, helps to break down individual barriers, with a focus on cooperation rather than competition, and so fosters opportunities for participants to develop group cohesiveness and pro‐social behaviour.

The participants are encouraged to step out of their comfort zone by challenge and adversity components of the course, such as rock climbing, high ropes, and expedition travel. Successful engagement in the programme can have positive peer effects within the programme. Successful behaviour may be observed by others, and perceived as something to aspire to.

Potentially important mediators suggested by this discussion are pro‐social behaviour and self‐worth. The quality of the relationship with the course counsellor may be an important moderator.

#### 1.3.3 The challenge element develops self‐worth from sense of achievement

Wilderness and adventure programs seek to tackle anti‐social behaviour through the challenge element thereby building self‐esteem and developing positive interpersonal skills, all of which may affect offending and reoffending.

New problem‐solving situations, drawing on different skills, are introduced to participants in a sequence of increasing difficulty—a process the Youth Hostel Association call ‘from zero to hero’. The challenges help the group members draw on their mental, emotional, and physical resources. Completion of such tasks leads to feelings of personal and social accomplishment. Programme facilitators provide coaching, teaching, support, nurturance, reinforcement, and encouragement supporting completion of the challenges during the course of the programme which reinforces this positive self‐development. In addition, the possibility to actively help another person via group work serves to increase one's self‐efficacy and self‐esteem (Yalom & Leszcz, 2005).

Potentially important mediators suggested by this discussion are social skills and self‐worth. The inclusion of a challenge element, or the degree of challenge involved, in a programme is thus a moderator for these outcomes, and so also the final outcome of offending.

#### 1.3.4 Diversion (time use)

Many youth programmes such as sports or after schools programmes have a diversion element since they provide the youth something to do rather than hang around in places where they are at risk of being drawn into criminal behaviour. This effect may also be there for wilderness, though their limited duration may limit or entirely obviate this effect.

Youth participating in the wilderness, therapy programme are away from their regular day to day environment and hence this prevents them from getting in trouble. However, the courses are of limited duration so this direct diversion effect will not be large. Some programmes may include a follow up component which seeks to continue to engage former participants, possibly with mentoring or repeat visits.

There may be a large indirect effect if the course leads them to develop an interest in an adventure sport, or even hiking, and so they get diverted from high‐risk activities. Participants may also be attracted to go to take a qualification related to outdoor activities, and later volunteer on, or be employed by, programmes such as they participated in.

Adventure learning interventions may be of a more sustained duration and so have a larger diversion effect, so duration is an important moderator.

Duration comprises three elements: session length, number of sessions and overall duration of the course. So, for example, a course may have full day sessions every weekend for two months. There are arguments both ways as to whether a more intense course (say, every day, for two weeks) will have a larger or smaller effect. On the one hand, greater intensity may enhance social bonding, but a less intense delivery over a longer period gives more time for relationships to develop, and for events to occur to participants which they bring back to discuss, thus allowing what some call a ‘progressive journey’.

#### 1.3.5 The benefits of the counselling component and mentoring

Wilderness therapy is facilitated by qualified professionals who may provide counselling support to participants either informally or through a formal counselling component. Required skills are technical skills related to tasks (canoeing, climbing etc), health and safety, and facilitation and therapeutic skills.

In addition to therapy, programme counsellors may play a mentoring role. This may be a formal part of the programme, and may continue after the engagement in wilderness or informal activities. But if not a formal part of the programme, a mentoring relationship may emerge.

Both the presence of a counselling element and the qualifications of the counsellor may be important moderators, as is space allowed in the programme for either formal or informal mentoring.

### 1.4 The theory of change diagram

**Figure 1:**
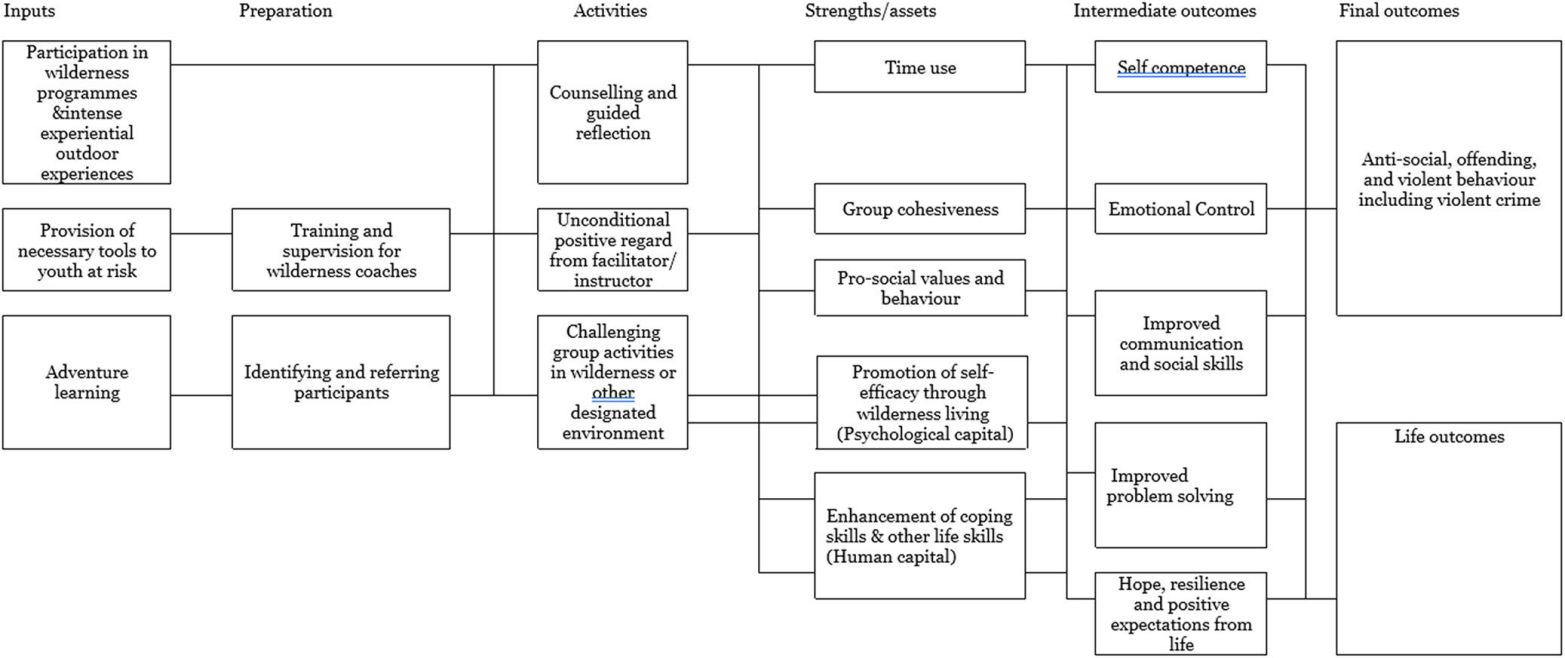
Theory of change diagram

The model, represented in Figure 1, explains how therapeutic wilderness, challenge activity and adventure learning programs lead to the outcomes such as reduced anti‐social behaviour, violence and offending behaviours.

The wilderness or adventure instructor, facilitator, coach or counsellor also plays a significant role in the process. Their role is to assure that this environment promotes growth by creating helping relationships which are genuine and congruent, and by providing unconditional positive regard.

The core of a wilderness therapy and adventure learning, as discussed above, involves intense experiential, interpersonal problem‐solving which is absent in the lives of many youth‐at‐risk. They lack appropriate role models and tools to develop the ability to articulate interpersonal problems, to conceive of options, to see the necessary means and potential obstacles, and to weigh consequences (Platt & Spivack, 1983).

The experience described by wilderness intervention or challenge activity provides the understanding and the tools, as well as the motivation, the support, and the reflection upon experience, necessary to learn these skills. Interpersonal problem‐solving may be a key to reducing asocial behaviour, not only in the wilderness, but in life.

This theory of change draws from the asset/strength‐based perspective. The strengths perspective pays attention to the resources of individuals that could potentially enable them to utilize these to cope with adversity than focusing only on the problems or deficits (Saleebey, 2000).

The interventions aim to tap and foster the assets and resources of children and adolescents (explained in Figure 1) and the helping process could eventually capacitate them to develop pro social behaviours.

This review will attempt to test the strength‐based theory and aims to provide insight into the processes and factors which contribute to positive outcomes in youth at risk through wilderness interventions (Table 1).

**Table 1:** Moderators from the theory of change analysis

Characteristic	Moderators
Type of activity	Physically demanding/challenge activity versus other
Counselling component	Contact activity versus other
	Group versus individual
	Indoor versus Outdoor
	Remote setting
	Including of therapeutic component
	Qualifications of counsellors
	Individual versus group counselling
Programme design	Programme length
	Programme intensity: number of sessions per week; number of hours per week
	Preparation for wilderness
	Aftercare and follow up (for longer run outcomes)
Age group	Age ranges
Sex	All male
	All female
	Mixed
	Not known
Offenders	CYP who have offended (desistance) versus those who have not (prevention)
Ethnicity	All or predominately minority ethnic group (80%+)
	Substantial minority ethnic group (30%–79%)
	No or minority of minority ethnic group (<30%)

### 1.5 Outcome measures

Primary outcomes: the primary outcomes are offending, violent and aggressive behaviour.

Secondary outcomes: secondary outcomes are intermediate outcomes identified in the theory of change, which include mental health and internalizing behaviour, self‐control, pro‐social behaviour and social skills, self‐worth, problem‐ solving skills.

As pointed out by Jolliffe and Farrington (2003), official records provide precise information (i.e., exact dates) about offenses; however, many of the specificities of offending can only be obtained through self‐reports (e.g., co‐offending, leaders and followers, motives, level of planning, etc.). Moreover, since not all crimes are detected, so self‐report may give a more accurate picture of crime levels (Kazemian & Farrington, 2005).

As a result, it is suggested that self‐reported and official measures of crime complement each other, and that each measure has specific strengths, and that combining them both compensates for each measure's shortcomings (Huizinga & Elliott, 1986).

All measures of offending will be combined such as self‐reported by youth and official records, but subgroup or moderator analysis will be considered (Table 2).

**Table 2:** Outcome category

	Examples
Offending Outcomes (Outcomes that refer to things that are against the law)	Violent offending (including weapon carrying), substance abuse, other offending and reoffending
Behavioural Outcomes (Outcomes that refer to the way in which someone acts)	Aggression; alcohol use/misuse; anti‐social and offending behaviour; externalizing behaviour; gang involvement and anti‐social peers; social skills and pro‐social behaviour; group membership and participation in community‐based activities (volunteering); time use
Psychosocial and cognitive outcomes (Psychosocial and cognitive)	Self‐esteem and self‐worth; mental health and resilience; self‐control and regulation (impulsivity)
Attitudes and beliefs (An attitude refer to how someone thinks or feels about something whereas a belief is an acceptance that something is true)	Pro‐social values: attitudes to aggression and use of violence; attitudes to crime and responses to crime (including drug use); attitudes to police and justice system and other authority
Family functioning/social support	family adult relationships; Quality of family relationships and family functioning, Improved interpersonal relationship with peers; social cohesion; safe spaces; engagement in education and academic achievement; practical life skills.

### 1.6 Cost effectiveness of wilderness, challenge and adventure learning programs

Evidence in general supports the view that prevention is more cost‐effective sending youth who offend to detention and correctional facilities. To test this view data are required on programme costs of alternative treatments as well as their benefits. We will review existing studies of cost effectiveness where available.

### 1.7 Why it is important to do this review

There is no recent effectiveness review of wilderness therapy.

The last review of wilderness therapy Wilson and Lipsey (2000) is very dated. The review found that ‘program length was not related to the magnitude of the effect on offending behaviour among the short‐ and medium‐term (less than 6 weeks) programs’ and ‘the duration variable acted as a proxy for some other characteristics of extended programs that account for their diminished effectiveness’. That review was published over 20 years ago and so is in need of an update.

There is a more recent review Fernee et al. (2017), but that is a qualitative review which explicitly excludes programmes intended for children displaying offending behaviour.

Bowen and Neill (2013) carried out a meta‐analytic review of outdoor challenge activities that included 197 studies. They found had a moderate short‐term effect size on behavioural outcomes, but did not report offending, and was not restricted to secondary and tertiary interventions.

There is no mixed methods systematic review that assesses the effects of wilderness therapy, and adventure learning on youth at risk of offending.

## 2 OBJECTIVES

The review will address the following research questions (RQs):

1. What are the long‐ and short‐term effects of wilderness therapy and adventure learning on anti‐social behaviour and violent and other offending behaviour?
  What factors explain any heterogeneity (i.e., moderate) these effects.
  What are the long‐ and short‐term effects of wilderness therapy and adventure learning on intermediate mental health and behaviour outcomes such as social skills and self‐regulation? What factors explain any heterogeneity (i.e., moderate) these effects? Factors such as setting (indoor/outdoor), quality of relationship with counsellors and the degree of the challenge element involved are important moderators of these effects, and help explain any observed heterogeneity across studies
2. What are the barriers and facilitators affecting the successful implementation of wilderness therapy and adventure learning programmes?
3. Are wilderness therapy and adventure learning interventions cost effective?

## 3 METHODS

### 3.1 Criteria for considering studies for this review

#### 3.1.1 Types of studies

Studies will be included in the review if they meet the following selection criteria:

- The programme involves a wilderness therapy, challenge activity or adventure leaning, all as described above.
- The programme is an organized activity
- The programme is targeted towards children and young people who have offended or are at risk of doing so who are aged 25 years or below (i.e., secondary and tertiary interventions only).

This is a mixed‐methods review that will include different study designs to address the different research questions (RQs). To evaluate the effectiveness of wilderness, challenge activities and adventure learning interventions (RQ 1 & 2), we will include:

∘ Experimental designs: randomized controlled trials.
∘ Non‐experimental designs: Designs with a non‐randomly assigned comparison group.

We will not include before versus after studies with no comparison group. Ex post single difference will be included as there is a comparison group.

Comparison group will be youth without any contact with the juvenile justice system, youth who are enroled in alternative types of treatment such as residential facilities and youth without an intervention. The nature of the comparison group will be a part of the moderator analysis.

Any comparison will be included, and the comparison condition will be coded. In addition, we will not combine active and passive comparisons in the same meta‐analysis. As part of the risk of bias assessment, we'll look at baseline balance, therefore studies with weak comparison groups will be labelled as low confidence.

We will use these evaluations to extract outcome data and conduct a meta‐analysis (or meta‐analyses) to evaluate the effectiveness of wilderness and adventure interventions, as well as moderators which explain observed variation in effects.

To understand the success factors and possible barriers to participation in wilderness and adventure learning interventions (RQ 3) we will include:

- Process evaluations and qualitative studies of interventions: Any evaluation or study of an eligible intervention discussing design and implementation issues.
- Information on barriers and facilitators will also be extracted from effectiveness studies if reported.

To evaluate the cost‐effectiveness of sports interventions (RQ 4), we include any other studies and reports presenting cost data, as well as extracting that information from effectiveness studies or process evaluations if available.

In the case of multi‐arm studies (ii) if there is a no treatment arm, that will be used as the comparison for all treatment arms; (ii) if there is an arm which is not eligible as wilderness/adventure therapy that arm will be designated as an active comparison condition; or (ii) if all arms are eligible treatments then the study will be used for moderator analysis but not used for average effect size estimates across all studies.

#### 3.1.2 Types of participants

Youth aged up to 25 years who have exhibited, or are deemed at risk of, anti‐social and offending behaviour. Young people with eating disorders and diagnosed psychiatric conditions, youth with a history of suicide ideation will be excluded from the review.

#### 3.1.3 Types of interventions

The review will include wilderness and adventure therapy programs.

Wilderness programmes are defined as follows:

(1) take place in ‘wilderness’ or nature setting;
(2) have an overnight stay element; and
(3) have an interpersonal element which may include group activities, or work with counsellors and therapists.

Adventure learning involves challenge‐based activities in which children and young people, usually in a group, have to overcome a challenge. The challenge may be in an outdoor setting—but need not be wilderness, it could be a local park—but may also be indoors. The challenge is intended to bring about change at a meta‐process level (behaviours, cognitions and unconscious processes that impede or support therapeutic change) (Itin, 2001).

In both cases the intervention must be targeted at youth who are at risk of offending, which includes those who have already offended. That is, we will include only secondary and tertiary interventions.

The intervention may take place in any setting (custody, community or school).

#### 3.1.4 Types of outcome measures

As pointed out by Jolliffe and Farrington (2003), official records provide precise information (i.e., exact dates) about offenses; however, many of the specificities of offending can only be obtained through self‐reports (e.g., co‐offending, leaders and followers, motives, level of planning, etc.). Moreover, since not all crimes are detected, so self‐report may give a more accurate picture of crime levels (Kazemian & Farrington, 2005).

As a result, it is suggested that self‐reported and official measures of crime complement each other, and that each measure has specific strengths, and that combining them both compensates for each measure's shortcomings (Huizinga & Elliott, 1986).

All measures of offending will be combined such as self‐reported by youth and official records, but subgroup or moderator analysis will be considered.

##### Primary outcomes

Primary outcomes: The primary outcomes are offending, violent and aggressive behaviour.

##### Secondary outcomes

Secondary outcomes: Secondary outcomes are intermediate outcomes identified in the theory of change, which include mental health and internalizing behaviour, self‐control, pro‐social behaviour and social skills, self‐worth, problem‐ solving skills.

#### 3.1.5 Types of settings

Adventure learning involves challenge‐based activities in which children and young people, usually in a group, have to overcome a challenge. The challenge may be in an outdoor setting—but need not be wilderness, it could be a local park—but may also be indoors. The challenge is intended to bring about change at a meta‐process level (behaviours, cognitions, and unconscious processes that impede or support therapeutic change) (Itin, 2001).

### 3.2 Search methods for identification of studies

#### 3.2.1 Electronic searches

We will use the following strategies to identify completed and on‐going potential studies:

Database: Medline, PsycInfo, PsycExtra, Social Policy & Practice, Scopus, Repec, ERIC, Econlit, CASE Engagement database (EEP, UCL), and the US National Criminal Justice.

Supporting Information: Appendix A presents an example of the search strings used for publication databases and search engines, with terms for interventions, regions and methodologies.

#### 3.2.2 Searching other resources

In addition to searching electronic databases, we will also screen the bibliographies of included studies and existing reviews of wilderness intervention programmes for eligible studies. Issues of relevant journals will also be hand‐searched for any possibly includable studies. A full list of these journals in provided below, as well as a list of research organizations and websites that we will search for any relevant publications (Table 3).

**Table 3:** List of journals

1	*Journal of Experiential Education*
2	*Journal of environment and behaviour*
3	*Journal of Research and Practice in Children's Services*
4	*Journal of creativity in mental health*
5	*Journal of child and Family studies*
6	*Child and Youth Care forum*
7	*Journal of therapeutic schools and programs*
8	*Journal of Contemporary Psychotherapy*
9	*Journal of Therapeutic Wilderness Camping*
10	*Journal of Youth and Adolescence*
11	*Journal of Leisurability*
12	*Journal of Mental Health Counseling*
13	*Journal of Adventure Education & Outdoor Learning*
14	*Journal of offender Rehabilitation*
15	*International Journal of offender Therapy and Comparative Criminology*
16	*Journal of offender Counseling, Services, and Rehabilitation*
17	*Therapeutic Recreation Journal*
18	*Canadian Journal of Criminology*
19	*Journal of emotional and behavioural disorders*
20	*Journal of experimental criminology*
21	*The open psychology journal*
22	*Australian journal of outdoor education*
23	*Journal of Behavior Technology Methods and Therapy*
24	*Journal of Child and Adolescent Group Therapy*
25	*Journal of Personality and Social Psychology*
26	*Juvenile and Family Court Journal*

In addition, we will the search relevant websites listed in Table 4. We will snowball to other websites identified in these searches, systematically documenting each website searched (website, URL, date, any filters or search strings used and studies identified for screening).

**Table 4:** List of websites

S. No	Webpage
1	The pine project https://pineproject.org/about/about-pine/
2	The Office of Juvenile Justice and Delinquency Prevention (OJJDP) https://ojjdp.ojp.gov/evidence-based-programs
3	Outward Bound https://www.outwardbound.org/about-us/history/
4	Wilderness Foundation UK https://wildernessfoundation.org.uk/
5	Aspiro adventure therapy https://aspiroadventure.com/about-us/mission/
6	Trails Carolina https://trailscarolina.com/
7	Blue ridge therapeutic wilderness https://blueridgewilderness.com/
8	Wingate Wilderness therapy https://www.wingatewildernesstherapy.com/
9	Bluefire Wilderness therapy https://bluefirewilderness.com/
10	True North Wilderness Programme https://truenorthwilderness.com/
11	Mountain Wise Wilderness Programme http://mountainwise.co.uk/wilderness-therapy.html

### 3.3 Data collection and analysis

#### 3.3.1 Selection of studies

The studies screening for inclusion/exclusion will be undertaken in two stages using EPPI reviewer 4 The first stage is title and abstract screening and the second is the screening of the full text. Both stages of screening will be done by two independent researchers using the screening tool. For T&A screening any study included by any screener will pass through to full text screening. Full text screening will be done by two independent researchers, with a third‐party arbitrator in case of disagreement. The screening tool included as Supporting Information: Appendix 3 is a mixed methods tool with both quantitative and qualitative data extraction codes. These also include extraction of barriers and facilitators as well as cost effectiveness of the studies.

#### 3.3.2 Data extraction and management

For impact and process evaluations/qualitative studies, we will use a standardized data extraction form (Supporting Information: Appendix 3) to extract data from all the studies that met our inclusion criteria. Data extraction from each study includes context/geographical information, population, study design and method, intervention types and outcomes type and subcategory. Two researchers will conduct the data extraction for each study. Both coders have been trained on the tool before starting. Disagreements will be resolved through discussion with a third reviewer consulted as needed.

#### 3.3.3 Assessment of risk of bias in included studies

The confidence in the study findings of all studies included in the review will be assessed using a critical appraisal tool for primary studies developed by Ashrita Saran, Ciara Keenan and Howard White. The tool has been constructed in such a manner that it covers both quantitative and qualitative studies. See Supporting Information: Appendix 4 for a version of the tool. Coding for critical appraisal will be carried out by two independent reviewers.

#### 3.3.4 Measures of treatment effect

Our study includes some outcomes which are typically reported as dichotomous variables (e.g., offending behaviour), and some which more often reported on the scale (e.g., behavioural measures). To perform the meta‐analysis we will use odds ratios for dichotomous variables and Hedge's *g* for continuous variables (as Hedge's *g* is preferred over Cohen's *d* for small samples which is expected to the case for this many studies included in this review).

Odds ratios will be computed via the available information for other effect sizes found in primary studies such as proportions, percentages, raw frequencies, regression coefficients, *χ* ^2^ and marginal distributions, etc. All effect size calculations will be performed using the Campbell online effect size calculator.

Where an effect, which is predominately reported as a dichotomous outcome, is reported in a paper as a continuous or ordinal measure then the effect size will be calculated as Hedge's *g*, and then converted to an odds ratio using OR = *e*(*g*/√3*π*).

Under a random effects model, analogue to the ANOVA approach will be used to match moderator analyses of a single categorical variable. Metanalytic regression techniques will be used to perform moderator analyses of continuous or multiple moderators, also under a random effects model.

All effect sizes will be reported in the common metric of odds ratios converted to a percentage reduction via 2 × 2 table for the purposes of communicating with policy makers and practitioners.

#### 3.3.5 Unit of analysis issues

The primary unit‐of‐analysis for the quantitative data within the studies of interest will usually be the individual, that is the specific youth within a programme. It is expected that these studies will report data at the programme level, reporting aggregate data for all youth in the programme.

Multiple papers or reports based on the same study or data will be treated as a single case for purposes of this review which fits with our proposed approached to mixed methods analysis, described below, in which the unit of analysis is the intervention (case), not the paper. That is, a paper report will only be treated as a separate case if the study sample does not include study participants included in any other coded study.

Where there are multiple papers, we will select the most complete reference, if all of the relevant information is available in a single source. But if the multiple reports each provide different information (e.g., different outcomes or different subgroups) then the data from all these reports will be coded as a single case.

#### 3.3.6 Criteria for determination of independent findings

A single study may report the same outcome multiple times for several reasons. We will treat such instances based on the reason for multiple reports as follows:

- Sub‐group analysis: We will code each sub‐group effect size as a unique effect along with details of the sub‐group for the purposes of moderator analysis. A code (full sample or sub sample) will be included so that only the full sample estimate is used in the overall meta‐analysis, but the appropriate sub‐sample estimate can be used for the sub‐group analysis.
- Follow up analysis: Where a study has outcome data on follow up, we will code all effects along with the time of the measure. These effect sizes will be used for an analysis of the durability of effects
- Model specification: Non‐experimental studies may report effect sizes with and without confounders. We will pick the effect size from the preferred model of the study authors (preferred would be the most parsimonious model which allows for confounders). If no preferred model is stated, then we will use the effect size from the most comprehensive model specification.

Intention to treat (ITT) versus treatment of the treated (ToT) outcome measures.

High attrition is a problem in many youth programmes. Differential attrition will be reported during the coding stage for all quantitative studies as it is one of the items in the critical appraisal tool.

Where attrition is high then it matters whether the reported effect size is ITT or ToT. Our intention is to report the meta‐analysis of ITT effects (adjusting ToT effects if necessary). This approach requires full reporting of losses to programme and losses to the sample, which are often not available.

#### 3.3.7 Assessment of heterogeneity

Heterogeneity between effect sizes studies will be assessed by reporting the Q‐value, degrees of freedom and the value of I2. Forest plots will be generated for visual representation of pooled effect size on both anti‐social behaviour and offending behaviour.

The causes of heterogeneity, if any, will be identified by visual inspection and moderator analysis. Separate forest plots will be presented for important moderators.

#### 3.3.8 Assessment of reporting biases

Publication‐selection bias will be assessed for the primary outcomes of anti‐social behaviour and offending behaviour by constructing a funnel plot for each of the two outcomes (Higgins et al., 2011). The funnel plot will be used for a trim‐and‐fill analysis and the calculation of Egger's test.

#### 3.3.9 Data synthesis

Carvalho and White (1997) identify various ways in which qualitative data may be used in an analysis of quantitative data. These ways are similar to those identified in the Cochrane Handbook which states that ‘qualitative evidence synthesis (commonly referred to as QES) can add value by providing decision makers with additional evidence to improve understanding of intervention complexity, contextual variations, implementation, and stakeholder preferences and experiences’ (Noyes et al., 2019).

This review adopts that approach, that is combining qualitative data with a quantitative meta‐analysis, within the framework of a theory‐based systematic review, TBSR (White, 2018). The TBSR approach, which has similarities with the framework synthesis approach (Carroll & Booth, 2015), takes the intervention as the unit of analysis, not the individual study. Different studies may contribute findings at different stages of the causal chain. For example, process evaluations and qualitative studies shed more light on implementation issues than do most effectiveness studies, such as the failure of a quality mentoring relationship to be established and why that was so, which can help explain both the size of, and variations in, effect sizes.

### 3.4 Treatment of qualitative research

Specifically, qualitative data can be (Carvalho & White, 1997):

- *Integrated* with quantitative data to elaborate the causal chain, that is the different causal mechanisms within the theory of change. For example, there may be a large gap between intention to treat and treatment of the treated effect sizes on account of high attrition as youth fail to show up in the first place or drop out. Qualitative data are usually best placed to understand barriers and facilitators to participation.
- Used to *confirm*, *enrich* and *illustrate* the findings of the quantitative analysis. For example, sports programmes may have both direct and indirect diversionary effects, which operate through time use, to reduce opportunities for criminal behaviour, and so to reduced criminal behaviour and police contact. Quotes from young people or their parents supporting these causal mechanisms add colour to the report, strengthening confidence in the effect as one that does operate through the posited causal mechanism.
- Used to *explain* study findings. The TBSR approach uses the funnel of attrition to recognize the fact that effect sizes get smaller moving along the causal chain from outputs and intermediate outcomes to final outcomes. The relevant factors in sport may include lack of participation for various reasons, weak links in the causal chain (e.g., qualitative studies highlight that young offenders may not lack self‐esteem, so the causal mechanism through higher self‐esteem through sports participation won't operate), the limited duration of programmes especially in the absence of opportunities for continued participation, and that sports may actually provide a channel for anti‐social behaviour and aggression. See Supporting Information: Annexure X for an illustration of the funnel of attrition.
- The previous point contains examples where qualitative data may contradict or *refute* the intended causal mechanisms, possibly leading to a counter‐theory (Carvalho & White, 2004), for example, that programmes for at risk youth may have iatrogenic effects but bringing them into contact with other anti‐social youth (Walsh et al., 2020).
- *Merged* with findings from quantitative analysis into a single set of implications for policy and practice.

The TBSR framework is shown in Table 5. Quantitative data are indicated as Qt and qualitative as Ql. Quantitative data refers to both effect sizes and factual quantitative data such as participation rates.

**Table 5:** Stages of the causal chain with data to be examined at each stage

Stage in causal chain	Data
Awareness of the programme amongst relevant service providers and target group	Know of programme, aware of eligibility criteria, purpose and how to access (Qt/Ql)
Enter the programme	Attrition (Qt)
Stay with programme for whole duration	Reasons do not participate or remain in programme (Ql)
Activities undertaken	Descriptive material (Ql)
Informal mentoring role	Mentoring relationship (Ql)
Diversion	Time use (Qt and Ql)
Connection to services	Channels for service connection (Ql)
Behavioural impact	Pro‐social behaviour. Self‐worth. Future outlook. (Qt supported by Ql).
Anti‐social behaviour and offending behaviour	Anti‐social behaviour, aggression, and criminal behaviour. Police contacts. (Qt)

Table 5 shows the TBSR framework which is used for both horizontal and vertical synthesis (White, 2018). In Table 6 an abbreviated version of the row headings from Table 5 are pivoted to become column headings. The data in Table 2 are subject to vertical, horizontal, and total synthesis.

**Table 6:** Theory based systematic framework

	Participation	Activities	Barriers and facilitators	Causal processes	Behavioural outcomes	Offending behaviour	
Case 1							Horizontal synthesis
Case 2							
‐‐‐							
Case n							
	Vertical synthesis						Overall synthesis

Vertical synthesis involves summarizing the evidence across all cases, which is the way systematic reviews are usually performed, especially for quantitative analysis of effects. In the case of qualitative data, vertical synthesis is a thematic analysis, in which common themes are identified across studies.

Horizontal synthesis summarizes across a case – which may be done in narrative reviews, but with the difference here that the data for an intervention may come from more than one study. The overall synthesis combines, both, though may well contain separate overall synthesis by sub‐group. The overall synthesis approach, drawing on both horizontal and vertical synthesis, ‘tells the story’ of if the intervention works, for whom, under what circumstances and why.

#### 3.4.1 Subgroup analysis and investigation of heterogeneity

Refer to (Table 1), in theory of change section.

In addition, we will also include as moderators (i) region of intervention (North America, Europe etc), (ii) publication type (i.e., published vs. unpublished); (iii) study design, and (iv) confidence in study findings (risk of bias).

Post hoc moderator analyses may be used depending on the analysis of patterns of heterogeneity in the data.

#### 3.4.2 Cost analyses

For the cost analysis in the review, we will extract data relating to costs from impact evaluations, process evaluations and cost related studies (cost effectiveness, cost‐analysis and studies that report cost estimates). The data may be an ingredients approach to listing intervention components and their cost, a cost effectiveness which includes an estimate of averted cases offending behaviour, or a cost–benefit analysis which sets costs against the financial savings from averted offending behaviour or later criminal activity.

The characteristics of these studies will be summarized narratively and in tables. To ensure comparability of cost‐estimates across studies the costs drawn from studies will be converted to British Pounds (GBP) and then to 2021 prices. However, the data of the estimate will also be shown as costs may change over time as intervention approaches change.

## CONTRIBUTIONS OF AUTHORS

- Content: Ashima Mohan, Howard White
- Systematic review methods: Ashima Mohan, Howard White
- Statistical analysis: Hannah Gaffney, Suchi Kapoor Malhotra, Monisha Laxminarayan, Howard White, Ashima Mohan
- Information retrieval: John Eyers

## DECLARATIONS OF INTEREST

Howard White was the CEO of the Campbell Collaboration at the time this protocol was written. As CEO he had no role in the editorial process.

## SOURCES OF SUPPORT

**Internal sources**

• No sources of support provided

**External sources**

• No sources of support provided

## Supporting information

Supporting information.

## Footnotes

^1^
The term adventure learning is also used to cover classroom‐based interventions in which CYP participate online in a real‐life expedition. These interventions are not targeted to at risk and offending youth, and their intended outcome is appreciation of different cultures and the environment, so they are not within our scope.

^2^

https://www.aee.org/what-is-experiential-education
