# Supplementary material for: PROTOCOL: The effectiveness of wilderness therapy and adventure learning in reducing anti‐social and offending behaviour in children and young people at risk of offending
Source: Campbell Syst Rev. 2022 Aug 31;18(3):e1270. doi: 10.1002/cl2.1270 (PMC9428765; doi:10.1002/cl2.1270)
Supplement: Supplementary file 1 — Supporting information. [file CL2-18-e1270-s001.docx]

# Appendices

## 1 Search Strategy

**Appendix 1**

1. **APA PsycInfo (Ovid) <1806 to January Week 3 2021> Searched 28^th^ January 2021**

1 (((wilderness or nature or outdoor or adventure*) adj3 (therap* or challeng* or program* or expedition* or experience* or adventure or camp or camps or camping)) or "outward bound").ti,ab. (7360)

2 "wilderness experience"/ or "adventure therapy"/ or therapeutic camps/ (796)

3 1 or 2 (7722)

4 "Adolescent Psychology"/ or exp Adolescent Attitudes/ or exp Early Adolescence/ or exp Adolescent Psychopathology/ or exp Adolescent Psychiatry/ (35678)

5 (adolescen* or teen* or youth or youths or juvenile* or "young people" or "young person*" or child* or delinquen* or anti-social or antisocial or "young offender*" or "young addict*" or ((disruptive or externali*) adj2 behavio*)).ti,ab. (930902)

6 behaviour change/ or exp behaviour disorders/ or exp aggressive behaviour/ or exp antisocial behaviour/ or exp behaviour problems/ or exp criminal behaviour/ or exp juvenile delinquency/ or juvenile justice/ (313314)

7 or/4-6 (1114059)

8 3 and 7 (**2319**)

1. **APA PsycExtra (Ovid) <1908 to December 09, 2020> Searched 28^th^ January 2021**

1 (((wilderness or nature or outdoor or adventure*) adj3 (therap* or challeng* or program* or expedition* or experience* or adventure or camp or camps or camping)) or "outward bound").ti,ab. (397)

2 "wilderness experience"/ or "adventure therapy"/ or therapeutic camps/ (72)

3 1 or 2 (437)

4 "Adolescent Psychology"/ or exp Adolescent Attitudes/ or exp Early Adolescence/ or exp Adolescent Psychopathology/ or exp Adolescent Psychiatry/ (2442)

5 (adolescen* or teen* or youth or youths or juvenile* or "young people" or "young person*" or child* or delinquen* or anti-social or antisocial or "young offender*" or "young addict*" or ((disruptive or externali*) adj2 behavio*)).ti,ab. (56591)

6 behaviour change/ or exp behaviour disorders/ or exp aggressive behaviour/ or exp antisocial behaviour/ or exp behaviour problems/ or exp criminal behaviour/ or exp juvenile delinquency/ or juvenile justice/ (27889)

7 or/4-6 (73305)

8 3 and 7 (**155**)

1. **Social Policy and Practice (Ovid) <202010> Searched 28^th^ January 2021**

1 (((wilderness or nature or outdoor or adventure*) adj3 (therap* or challeng* or program* or expedition* or experience* or adventure or camp or camps or camping)) or "outward bound").ti,ab. (451)

2 ecotherapy.de. (91)

3 or/1-2 (510)

4 (adolescen* or teen* or youth or youths or juvenile* or "young people" or "young person*" or child* or delinquen* or anti-social or antisocial or "young offender*" or "young addict*" or ((disruptive or externali*) adj2 behavio*)).ti,ab. (101142)

5 ("young people" or children).de. (50327)

6 or/4-5 (107917)

7 3 and 6 (**225**)

1. **Web of Science (Social Sciences Citation Index & Arts & Humanities Citation Index) – Searched 28^th^ January 2021**

**Also Web of Science Conferences & Proceedings (SSCI/AHCI) - Searched 29th January 2021 - 172**

# 3 **1,558**

#2 AND #1

Indexes=SSCI, A&HCI Timespan=1970-2021

# 2 893,929

TS=((adolescen* or teen* or youth or youths or juvenile* or "young people" or "young person*" or child* or delinquen* or anti-social or antisocial or "young offender*" or "young addict*" or ((disruptive or externali*) NEAR/2 behavio*)))

Indexes=SSCI, A&HCI Timespan=1970-2021

# 1 15,640

TS=(((wilderness or nature or outdoor or adventure*) NEAR/3 (therap* or challeng* or program* or expedition* or experience* or adventure or camp or camps or camping) ) or "outward bound")

Indexes=SSCI, A&HCI Timespan=1970-2021

1. **ERIC (Ebsco) – Searched 28^th^ January 2021**

S7  S5 AND S6

Database - ERIC  **2,175**

S6  S3 OR S4

 388,721

S5  S1 OR S2

 9,078

S4  (DE "Early Adolescents" OR DE "Late Adolescents" OR DE "Youth" OR DE "Disadvantaged Youth" OR DE "Out of School Youth" OR DE "Rural Youth" OR DE "Urban Youth") OR DE "Delinquency" OR DE "Delinquency Prevention" OR DE "Delinquent Rehabilitation" OR DE "Juvenile Gangs" OR DE "Juvenile Justice" OR DE "Recidivism" OR DE "Youth Problems")

 38,864

S3  TI ( (adolescen* or teen* or youth or youths or juvenile* or "young people" or "young person*" or child* or delinquen* or anti-social or antisocial or "young offender*" or "young addict*" or ((disruptive or externali*) N2 behavio*)) ) OR AB ( (adolescen* or teen* or youth or youths or juvenile* or "young people" or "young person*" or child* or delinquen* or anti-social or antisocial or "young offender*" or "young addict*" or ((disruptive or externali*) N2 behavio*)) )

 379,737

S2  DE "Field Experience Programs" OR DE "Adventure Education" OR DE "Resident Camp Programs"

 7,224

S1  TI ( (((wilderness or outdoor or adventure*) N3 (therap* or challeng* or program* or expedition* or experience* or adventure or camp or camps or camping)) or "outward bound") ) OR AB ( (((wilderness or outdoor or adventure*) N3 (therap* or challeng* or program* or expedition* or experience* or adventure or camp or camps or camping)) or "outward bound") )

 3,108

1. **Repec from Ebsco Discovery – Searched 21st January 2021**

S3  S1 AND S2

Database - Discovery Service for 3ie, Inc.

16,311 – **Limited to Repec - 93**

S2  TI ( ( (((wilderness or outdoor or adventure* or nature) N3 (therap* or challeng* or program* or expedition* or experience* or adventure or camp or camps or camping)) or "outward bound") ) ) OR AB ( ( (((wilderness or outdoor or adventure* or nature) N3 (therap* or challeng* or program* or expedition* or experience* or adventure or camp or camps or camping)) or "outward bound") ) ) OR SU ( ( (((wilderness or outdoor or adventure* or nature) N3 (therap* or challeng* or program* or expedition* or experience* or adventure or camp or camps or camping)) or "outward bound") ) )

 114,558

S1  TI ( ( (adolescen* or teen* or youth or youths or juvenile* or "young people" or "young person*" or child* or delinquen* or anti-social or antisocial or "young offender*" or "young addict*" or ((disruptive or externali*) N2 behavio*)) ) ) OR AB ( ( (adolescen* or teen* or youth or youths or juvenile* or "young people" or "young person*" or child* or delinquen* or anti-social or antisocial or "young offender*" or "young addict*" or ((disruptive or externali*) N2 behavio*)) ) ) OR SU ( ( (adolescen* or teen* or youth or youths or juvenile* or "young people" or "young person*" or child* or delinquen* or anti-social or antisocial or "young offender*" or "young addict*" or ((disruptive or externali*) N2 behavio*)) ) )

 13,547,295

## 2 List of journals and websites to be hand-searched

[EnteList of the Journals
1 Journal of Experiential Education
2 Journal of environment and behaviour
3 Journal of Research and Practice in Children's Services
4 Journal of creativity in mental health
5 Journal of child and Family studies
6 Child and youthcare forum
7 Journal of therapeutic schools and programs 
8 Journal of Contemporary Psychotherapy
9 Journal of Therapeutic Wilderness Camping
10 Journal of Youth and Adolescence
11 Journal of Leisurability
12 Journal of Mental Health Counseling
13 Journal of Adventure Education & Outdoor Learning
14 Journal of offender Rehabilitation
15 International Journal of offender Therapy and Comparative Criminology
16 Journal of offender Counseling, Services, and Rehabilitation
17 Therapeutic Recreation Journal
18 Canadian Journal of Criminology
19 Journal of emotional and behavioural disorders
20 Journal of experimental criminology
21 The open psychology journal
22 Australian journal of outdoor education
23 Journal of Behaviour Technology Methods and Therapy
24 Journal of Child and Adolescent Group Therapy
25 Journal of Personality and Social Psychology
26 Juvenile and Family Court Journal

List of Websites

| S. No | Webpage |
| --- | --- |
| 1 | The pine project   https://pineproject.org/about/about-pine/ |
| 2 | The Office of Juvenile Justice and Delinquency Prevention (OJJDP)  <https://ojjdp.ojp.gov/evidence-based-programs> |
| 3 | Outward Bound  https://www.outwardbound.org/about-us/history/ |
| 4 | Wilderness Foundation UK  https://wildernessfoundation.org.uk/ |
| 5 | Aspiro adventure therapy  https://aspiroadventure.com/about-us/mission/ |
| 6 | Trails Carolina  https://trailscarolina.com/ |
| 7 | Blue ridge therapeutic wilderness  https://blueridgewilderness.com/ |
| 8 | Wingate Wilderness therapy  https://www.wingatewildernesstherapy.com/ |
| 9 | Bluefire Wilderness therapy  https://bluefirewilderness.com/ |
| 10 | True North Wilderness Programme  https://truenorthwilderness.com/ |
| 11 | Mountain Wise Wilderness Programme  http://mountainwise.co.uk/wilderness-therapy.html |

## 3 Screening Tool

| Q1 | Language | Is the paper in english | No-exclude |
| --- | --- | --- | --- |
|  |  |  | yes-include |
|  |  |  |  |
| Q2 | Publication Date | Published after 1970 | No-exclude |
|  |  |  | Yes-include |
|  |  |  |  |
| Q3 | Population | Is the population eligible for the intervention youth up to the age of 25 who display, or at are risk of displaying, anti-social or offending behaviour | No-exclude |
|  |  |  | Yes-include |
|  |  |  |  |
| Q4 | Intervention | is the intervention (a) involving a physical challenge in the wilderness and (b) with an overnight component | Yes-include |
|  |  |  | No-exclude |
|  |  |  |  |
| Q5 | Outcomes | is the main outcome of the study anti-social behaviour and offending behaviour? | No-exclude |
|  |  |  | Yes-include |
|  |  |  |  |
| Q6 | Types of Studies | Are the studies Experimental and non-experimental designs with comparisonm group, instrument variables and interrupted time series? | No-exclude |
|  |  |  | yes-include |

## 4 Coding Tool

**Appendix 4 Coding Tool**

| **Category** | **Sub Category** |
| --- | --- |
| **Publication Status** | - Ongoing - Completed |
| **Region** | - East Asia & Pacific - Europe & Central Asia - Latin America & Caribbean - Middle East & North Africa - South Asia - Sub Saharan Africa - America - Not mentioned |
| **Country** |  |
| **Countries by income** | - Lower- Middle Income Countries - Low- Income Countries - Upper- Middle Income Countries |
| **Settings** | - Rural - Urban - Rural and Urban (Both) - Indoor - Outdoor - Not clear |
| **Name of the project/ intervention**  **Type of activity** |  |
| **Funding agency** |  |
| **Duration of Intervention** | - Less than 6 months - 6 months-1 year - 1-2 years - 2-3 years - More than 3 years - Not mentioned |
| **Frequency of meetings** | - More than once a week - Once a week - 2-3 times a month - Once a month - Less than one a month - Not clear |
| **Length of meetings** | - Less than one hour - Approximately one hour - 1-2 hours - Over 2 hours - Not clear |
| **Unit of delivery** | - Individual-One to one - Group |
| **Age** | - under 9 - 10-14 - 15-17 - 18-25 |
| **Gender** | - Male - Female - Non-Binary - Both - Not reported |
| **BAME** | - Mainly/exclusively (80%) - Partly - None - Not clear |
| **Study Design** | - Experimental design - Non- experimental design - Process evaluation or qualitative intervention study - Mixed method - Cost analysis |
| **Sample Size** | - Less than 100 - 100-300 size - More than 300 - Not mentioned |
| **Costs involved** | - Training - Infrastructure - Salary - Monitoring & supervision - Other - Not mentioned |
| **Intervention category** | **Intervention sub-category (for structured approaches)** |
| **Behavioural interventions** | - Mental health & therapeutic interventions - Social and emotional interventions - Alcohol and drug related interventions |
| **Skill development/teaching** | - Academic support/Remedial coaching - Skill development (communication skills, leadership skills, social skills, coping skills, life skills etc) - Career /vocational guidance& support - Providing other desired information & guidance |
| **Outcome Domain** | **Outcome Sub-domain** |
| **Offending related outcomes** | - Violence - Crime/ anti-social activities - Gang membership - Recidivism |
| **Child-centred** | - Attitudes and belief (self-concept, esteem, confidence etc) - Mental health, internalizing behaviour and self-regulation, externalizing and risk-taking behaviour - Substance use - Social outcomes & emotional outcomes (improvement in interpersonal relationships, communication, improved adjustment etc) - Cognitive development- Social Cognition and pro social behaviour - Identity Development - Improved Psycho- social functioning & wellbeing. |
| **Family & Peers** | - Quality of family relationships and family functioning - Improved interpersonal relationship with peers |
| **School related outcomes** | - Improvement in interpersonal relationships in the school environment. - Improvement in academic performance - Improvement in school engagement |
| **Cost Effectiveness** | Open coding |

## 5 Effect size coding

| Effect size | Numerical entry |
| --- | --- |
| Outcome name | Open coding |
| Outcome domain | For each outcome coded |
| Type of effect | Difference in means  Difference in proportions  Regression coefficient  Odds ratio  Risk ratio |
| Sub group analysis | Whole sample  Sub-group (name) |
| Duration | Endline  Post endline (duration) |
| Sample sizes | For each effect coded, and corresponding control sample size |
| Standard deviations | For each outcome coded |
| Treatment effect | Intention to treat  Treatment of treated |

## 6 Critical Appraisal tool

| **Appendix 5 Critical appraisal tool for primary studies: effectiveness** | | | |
| --- | --- | --- | --- |
| *Item* | **Description** | **Key** | **Notes** |
| *Intervention* | Is the intervention clearly named and described, including all relevant components. See examples below. | High: full and clear description, so that the main components and how they are delivered are clear  Medium: Partial description  Low: Little or no description |  |
| *Evaluation questions* | Are the evaluation questions clearly stated? | High: full and clear description, so that the main components and how they are delivered are clear  Medium: Partial description  Low: Little or no description |  |
| *Study design* | Use the study design coding | High: Experimental  Medium: Non-experimental  Low: Before versus after |  |
| *Outcomes* | Are the outcomes clearly defined? Where appropriate do they use an existing, validated measurement tool?  See examples below. | High: full and clear definition using validated instruments where available (a researcher wishing to use these outcomes would have sufficient information to do so)  Medium: Partial definition. May use validated instruments but without sufficient references to source.  Low: Little or no definition |  |
| *Sample size (power calculation)* | Do the authors report a power calculation as the basis for sample size? | High: Power calculation report and sample size meets necessary sample size  Medium: Power calculation mentioned and sample size meets necessary sample size  Low: No mention of power calculation. |  |
| *Attrition* | Reported for endline and longest follow up.  Calculate overall attrition and differential attrition (see example below). It is often necessary to calculate from table of results. If sample size varies by outcome calculate for highest attrition. | High: Attrition within IES conservative standard  Medium: Attrition within IES liberal standard  Low: Attrition outside IES liberal standard |  |
| *Baseline Equivalence* | A test that the average values of outcomes and confounders are the same in treatment and comparison at baseline. Sampling error means we'd expect 5% of tests to appear as significantly different even if no true population difference. Properly done randomization should, with sufficient sample size, ensure balance but it is not guaranteed | High: baseline balance on 90% or more of reported indicators    Medium: baseline balance on 80% or more of reported indicators OR RCT with randomization over at least 20 clusters or 50 individuals and no baseline balance table reported.    Low: Baseline balance not reported OR balance on less than 80% of reported indicators OR ex-post single difference design with significant difference in baseline outcomes. |  |
| *Overall (including questions for all studies)* | The overall score uses the weakest link in the chain principle i.e., is the lowest score on any item | High: High on all items  Medium: No lower than medium on any item  Low: At least one low |  |

**Questions for process evaluations (apply to implementation sections) [used for any study coded as having implementation evidence]**

|  |  | **High** | **Medium** | **Low** |  | **Low** |
| --- | --- | --- | --- | --- | --- | --- |
| *1* | Is the qualitative methodology described? | Yes |  | No | >> 3 |  |
| *2* | Is the qualitatively methodology appropriate to address the evaluation questions? | Yes | Partially | No |  | Insufficient detail |
| *3* | Is the recruitment or sampling strategy described? | Yes |  | No | >> 5 |  |
| *4* | Is the recruitment or sampling strategy appropriate to address the evaluation questions? | Yes | Partially | No |  | Insufficient detail |
| *5* | Are the researcher's own position, assumptions and possible biases outlined? | Yes | Partially | No |  |  |
| *6* | Have ethical considerations been sufficiently considered? | Yes | Partially | No |  | Insufficient detail |
| *7* | Is the data analysis approach adequately described? | Yes |  | No | >>9 |  |
| *8* | Is the data analysis sufficiently rigorous? | Yes | Partially | No |  |  |
| *9* | Are the implications or recommendations clearly based in the evidence from the study? | Yes | Partially | No |  |  |
| *10* | Overall (including questions for all studies- The overall score uses the weakest link in the chain principle i.e., is the lowest score on any item | High: High on all items  Medium: No lower than medium on any item  Low: At least one low |  |  |  |  |
